# The Effect of Examined Lymph Nodes and Lymph Node Ratio on Pathological Nodal Classification in the Lung Adenosquamous Carcinoma After Lobectomy

**DOI:** 10.3389/fsurg.2022.909810

**Published:** 2022-06-09

**Authors:** Shoujie Feng, Xiangming Liu, Bing Huang, Jing Shi, Hao Zhang

**Affiliations:** ^1^ Department of Thoracic Surgery, Affiliated Hospital of Xuzhou Medical University, Xuzhou, China; ^2^ Thoracic Surgery Laboratory, Xuzhou Medical University, 84 West Huaihai Road, Xuzhou, China; ^3^ Department of Thoracic Surgery, Affiliated Huaihai Hospital of Xuzhou Medical University, Xuzhou, China; ^4^ Department of Radiology, Affiliated Huaihai Hospital of Xuzhou Medical University, Xuzhou, China

**Keywords:** adenosquamous carcinoma, non-small cell lung cancer, lymph node, lymph node ratio, survival

## Abstract

**Objective:**

The effects of examined lymph nodes (LNs) and lymph node ratio (LNR) on pN classification and the prognosis are unclear in lung adenosquamous carcinoma (ASC) patients. Thus, this study aimed to investigate the significance of LNs and LNR in the prognosis of ASC and the impact of the abovementioned factors on the pN classification.

**Methods:**

Patients diagnosed with pathological stage T1-4N0-2M0 ASC from the Surveillance Epidemiology and End Results database were included in the study. The primary clinical endpoint was cancer-specific survival (CSS). The optimal cutoff values of the LNs and LNR were determined. An LN indicator, including pN0 #LNs ≤9, pN0 #LNs >9, pN^+^ #LNR ≤0.53, and pN^+^ #LNR > 0.53, was developed. Concordance index (C-index) was used to compare the prognostic predictive ability between N classification and LN indicator. The univariable and multivariable Cox regression analyses were used in this study.

**Results:**

The cohort of 1,416 patients were included in the study. The level of LNs stratified the patients without metastasis of lymph nodes (pN0 #LNs ≤9 vs. pN0 #LNs >9, unadjusted hazard ratio [HR] = 1.255, *P* = 0.037). Two groups based on the cutoff value of LNR differentiated prognosis of patients with metastasis of lymph nodes (pN^+^ #LNR >0.53 vs. pN^+^ #LNR ≤0.53, unadjusted HR = 1.703, *P* = 0.001). The LN indicator had a much better predictive ability over N classification in this cohort (LN indicator: C-index = 0.615; N classification: C-index = 0.602, *P* = 0.001).

**Conclusions:**

We explored clinicopathological factors affecting prognosis in resected lung ASC patients. Besides, the LN indicator was confirmed to be played an essential role in affecting the survival rate in ASC patients. The high-level LNs or low-level LNR might be corelated to improved survival outcomes.

## Introduction

Lung cancer is the second incidence and the first mortality diseases in the cancer spectrum worldwide (1), which is mainly classified two histological types, including non-small cell lung cancer and small cell lung cancer (2). The 5-year overall survival rates of non-small cell lung cancer and small-cell lung cancer are about 23% and 6%, respectively. Of note, the prognosis of small-cell lung cancer is poor (3). The adenocarcinoma and squamous cell carcinoma, as common pathological types in the lung cancer, were studied by many researchers (4–8). Adenosquamous carcinoma (ASC) accounts for about 3% in the non-small cell lung cancers according to a previous report (9). The prognosis of ASC is the worst among ASC, adenocarcinoma, and squamous cell carcinoma (9). Surgery is a key method for the treatment of lung cancer, which could effectively improve the survival outcomes of resected lung cancer (2, 10). However, the research about the postoperative prognosis of ASC was lacking. Therefore, it is important to explore the factors affecting postoperative survival of ASC.

The examined lymph nodes (LNs) and lymph node ratio (LNR) were confirmed as the significant prognostic indicators for resected non-small cell lung cancers based on previous studies (11–14). LNR was defined as the ratio of the number of metastatic lymph nodes divided by the total number of dissected lymph nodes. However, for ASC patients, the effects of LNs and LNR on nodal classification and the prognosis are unclear. Thus, this study was aimed to investigate the significance of LNs and LNR in prognosis of ASC and the impact of the abovementioned factors on the nodal classification.

## Methods and Materials

### Patients

**Figure 1:**
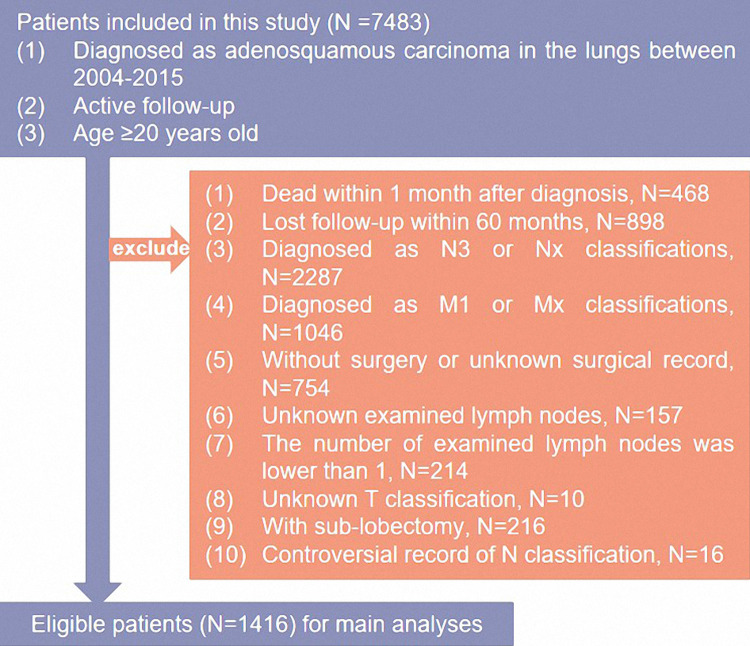
The flowchart of the study.

The cases were collected from the Surveillance, Epidemiology, and End Results database by a software, SEER*Stat 8.3.9. (seer.cancer.gov/seerstat). Eligible patients for main analyses met the following criteria: (1) pathologically diagnosed as ASC lung cancer; (2) patients with virtual survival status and clear survival time; (3) diagnosed between 2000–2018 and active follow-up; (4) underwent lobectomy and dissection of lymph nodes. Then, the tumor (T), nodes (N), and metastasis (M) stages were reassigned according to the 8th American Joint Committee on Cancer (15). Patients were excluded if they: (1) were diagnosed with N3 or M1 diseases; (2) had unknown resected or positive lymph nodes; (3) had unknown T classification or surgery type. The detailed information about selection standards is shown in Figure 1.

### Follow-up

The follow-up information on this cohort was updated in November 2020. The median follow-up time was 46.0 months. The time interval between the operation of the primary tumor and the cancer-caused mortality was defined as cancer-specific survival (CSS). Cases were censored at the end of follow-up. CSS was considered best concerning for clinical relevance.

### Statistical Analyses

Univariable and multivariable Cox regression analyses were performed to calculate the hazard ratio (HR) and 95% confidence interval (CI) of the variables for cancer-specific mortality. Those factors included sex, age, race, marital status, tumor location, surgical approach, chemotherapy, radiotherapy, grade, pathological T (pT) classification, pathological N (pN) classification, LNR, and LNs. A two-sided *P* < 0.05 was defined as statistically significant. Survival curves were generated through Cox regression analysis. For evaluating the effect of LNR and LNs on the pN classification, we calculated the cutoff values of LNs and LNR in the cohort with pN0 classification and pN+ classification, respectively. Concordance index (C-index) was used R 4.1.2 software (“compareC” packages) to compare the prognostic predictive ability between N classification and LN indicator. Standard error (SE) was performed to evaluate the stability of C-index. The optimal cutoff points of LNs and LNR were calculated by the “survminer” and “survival” packages in R 4.1.2 software (https://www.r-project.org/), respectively. Other analyses were performed using software SPSS 25.0 (IBM SPSS, Inc., Chicago, IL, USA).

## Results

### Patient Characteristics

**Figure 2:**
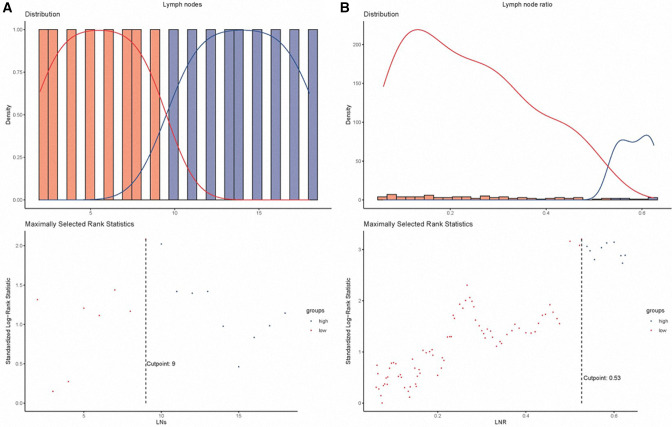
The cutoff value processing for lymph nodes (**A**) and lymph node ratio (**B**). LNs, lymph nodes; LNR, lymph node ratio.

A total of 1,416 patients entered main analyses. In this study, men outnumbered women, constituting 53.8% of the patients. 436 (30.8%) patients were age 64 and below, whereas 980 (69.2%) were over 64 years old. The majority of patients were diagnosed with the poor-undifferentiated grade, comprising more than 50% of the patients. In terms of the pN classification, most patients were diagnosed with classification N0 (*N* = 954, 67.4%). The proportion of patients who did not undergo radiotherapy was high, reaching 86.3%. Patients with classification pT1 accounted for 35.7% (*N* = 506) in this cohort. The median LNs was 8 (range 1–83). The cutoff points of LNs and LNR were 9 and 0.53, respectively (Figure 2). We further combined the pN classification, LNs, and LNR into a LN indicator. The number of patients with N0 #LNs ≤9 was 594 (41.9%). Table 1 presents the baseline characteristics of the entire cohort.

**Table 1:** The characteristics of adenosquamous carcinoma patients.

Total		LN indicator			
		pN0 #LNs >9	pN0 #LNs ≤9	pN^+^ #LNR ≤0.53	pN^+^ #LNR >0.53
		*N* = 360	*N* = 594	*N* = 396	*N* = 66
Sex	Male	197 (25.9%)	311 (40.8%)	214 (28.1%)	40 (5.2%)
	Female	163 (24.9%)	283 (43.3%)	182 (27.8%)	26 (4.0%)
Age	<65	94 (21.6%)	185 (42.4%)	135 (31.0%)	22 (5.0%)
	>64	266 (27.1%)	409 (41.7%)	261 (26.6%)	44 (4.5%)
Race	Caucasians	316 (26.2%)	510 (42.2%)	329 (27.2%)	53 (4.4%)
	Other	44 (21.2%)	84 (40.4%)	67 (32.2%)	13 (6.3%)
Surgery	Lobectomy	335 (25.6%)	584 (44.5%)	335 (25.6%)	57 (4.3%)
	Pneumonectomy	25 (23.8%)	10 (9.5%)	61 (58.1%)	9 (8.6%)
Radiotherapy	None	342 (28.0%)	548 (44.8%)	293 (24.0%)	39 (3.2%)
	Yes	17 (9.1%)	45 (24.2%)	98 (52.7%)	26 (14.0%)
	Unknown	1 (12.5%)	1 (12.5%)	5 (62.5%)	1 (12.5%)
Chemotherapy	None	300 (30.3%)	499 (50.4%)	162 (16.4%)	29 (2.9%)
	Yes	60 (14.1%)	95 (22.3%)	234 (54.9%)	37 (8.7%)
Marital status	None	125 (23.7%)	234 (44.4%)	146 (27.7%)	22 (4.2%)
	Yes	226 (26.7%)	348 (41.0%)	234 (27.6%)	40 (4.7%)
	Unknown	9 (22.0%)	12 (29.3%)	16 (39.0%)	4 (9.8%)
Grade	I-II	142 (27.2%)	245 (46.9%)	113 (21.6%)	22 (4.2%)
	III-IV	193 (24.3%)	306 (38.5%)	260 (32.7%)	36 (4.5%)
	Unknown	25 (25.3%)	43 (43.4%)	23 (23.2%)	8 (8.1%)
pT classification	T1	150 (29.6%)	251 (49.6%)	92 (18.2%)	13 (2.6%)
	T2a	103 (24.8%)	177 (42.5%)	117 (28.1%)	19 (4.6%)
	T2b	42 (25.0%)	59 (35.1%)	58 (34.5%)	9 (5.4%)
	T3	35 (22.4%)	49 (31.4%)	60 (38.5%)	12 (7.7%)
	T4	30 (17.6%)	58 (34.1%)	69 (40.6%)	13 (7.6%)
pN classification	N0	360 (37.7%)	594 (62.3%)	0 (0.0%)	0 (0.0%)
	N1	0 (0.0%)	0 (0.0%)	224 (88.2%)	30 (11.8%)
	N2	0 (0.0%)	0 (0.0%)	172 (82.7%)	36 (17.3%)

*LN, lymph node; LNR, lymph node ratio; pT, pathological T; pN, pathological N.*

### Prognostic Significance of LN Indicator

**Figure 3:**
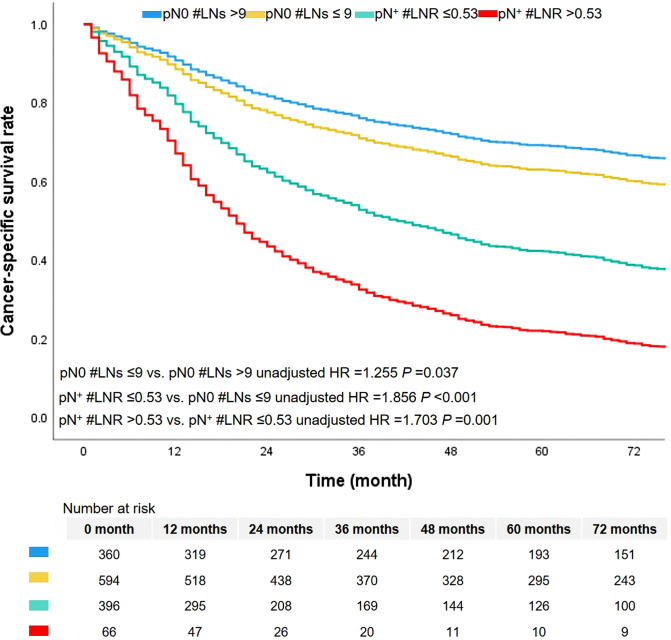
The survival curves based on LN indicator. LN, lymph node.

In this cohort, there were 672 cases dead due to ASC. The median survival time was 46.0 months, ranging from 1.0 to 179.0 months. The 1-year, 3-year, and 5-year CSS rates were 74.0%, 61.0%, and 54.0%, respectively. The level of LNs stratified the patients without metastasis of lymph nodes (pN0 #LNs ≤9 vs. pN0 #LNs >9, unadjusted HR = 1.255, 95% CI, 1.013–1.554, *P* = 0.037, Figure 3). The 5-year CSS rates of patients with pN0 #LNs ≤9 or pN0 #LNs >9 were 41.0% and 50.0%, respectively. Two groups based on the cutoff value of LNR differentiated prognosis of patients with metastasis of lymph nodes (pN^+^ #LNR >0.53 vs. pN^+^ #LNR ≤0.53, unadjusted HR = 1.703, 95% CI, 1.260–1.303, *P* = 0.001, Figure 3). Besides, the patients with pN^+^ #LNR >0.53 had a lower 5-year CSS rate than cases with pN^+^ #LNR ≤0.53 (11.0% vs. 26.0%).

### Univariable and Multivariable Analyses

The outcomes of univariable and multivariable analyses were presented in Table 2. In order to discriminate the prognostic factors, a total of 11 variables were included in the univariable Cox regression analysis. The characteristics of female, older age, lower pT classification, high grade, and lobectomy were considered to improve the survival outcomes. Some variables including race and marital status had no significant influence on survival. Furthermore, multivariable analysis confirmed age, pT classification, chemotherapy, and LN indicator (all *P* < 0.05) as independent prognostic factors after eliminating confounding factors.

**Table 2:** Univariable and multivariable analyses for cancer-specific mortality.

	Univariable analysis			Multivariable analysis		
	HR	95% Cl	*P*-value	HR	95% Cl	*P*-value
Sex						
Male	1	reference		1	reference	
Female	0.850	0.730–0.989	0.035	0.877	0.752–1.022	0.092
Age (years)						
<65	1	reference		1	reference	
>64	1.333	1.127–1.576	0.001	1.397	1.174–1.662	<0.001
Location						
Upper lobe	1	reference		1	reference	
Middle lobe	1.153	0.815–1.630	0.422	1.262	0.889–1.791	0.193
Lower lobe	1.272	1.081–1.499	0.004	1.131	0.957–1.336	0.149
Other	1.526	0.973–2.393	0.065	1.045	0.653–1.672	0.854
Unknown	0.931	0.415–2.086	0.861	1.013	0.446–2.301	0.974
pT classification						
T1	1	reference		1	reference	
T2a	1.575	1.286–1.930	<0.001	1.475	1.200–1.813	<0.001
T2b	1.681	1.293–2.185	<0.001	1.513	1.156–1.981	0.003
T3	2.358	1.836–3.028	<0.001	2.115	1.635–2.735	<0.001
T4	3.943	3.122–4.978	<0.001	3.250	2.514–4.200	<0.001
Grade						
I–II	1	reference		1	reference	
III–IV	1.267	1.077–1.491	0.004	1.155	0.980–1.363	0.086
Unknown	0.921	0.660–1.285	0.628	0.816	0.582–1.143	0.237
Surgery Approach						
Lobectomy	1	reference		1	reference	
Pneumonectomy	2.265	1.761–2.913	<0.001	1.155	0.980–1.363	0.086
Chemotherapy						
No	1	reference		1	reference	
Yes	1.280	1.092–1.500	0.002	0.717	0.586–0.876	0.001
Radiation						
No	1	reference		1	reference	
Yes	1.728	1.417–2.106	<0.001	1.214	0.958–1.537	0.107
Unknown	1.652	0.618–4.420	0.317	1.400	0.518–3.783	0.507
LN indicator						
pN0 #LNs ≤9	1	reference		1	reference	
pN0 #LNs >9	1.255	1.013–1.554	0.037	1.288	1.038–1.598	0.022
pN^+^ #LNR ≤0.53	2.338	1.884–2.902	<0.001	2.160	1.711–2.729	<0.001
pN^+^ #LNR >0.53	4.112	2.971–5.691	<0.001	3.672	2.613–5.160	<0.001
Race						
Caucasians	1	reference				
Others	1.077	0.874–1.327	0.488			
Marital status						
None	1	reference				
Married	0.927	.792–1.084	0.340			
Unknown	0.726	0.416–1.268	0.261			

*HR, hazard ratio; CI, confidence interval; LN, lymph node; LNR, lymph node ratio; pT, pathological T; pN, pathological N.*

*The method of Cox regression was “Enter selection”.*

### The Comparison Between pN Classification and LN Indicator

**Figure 4:**
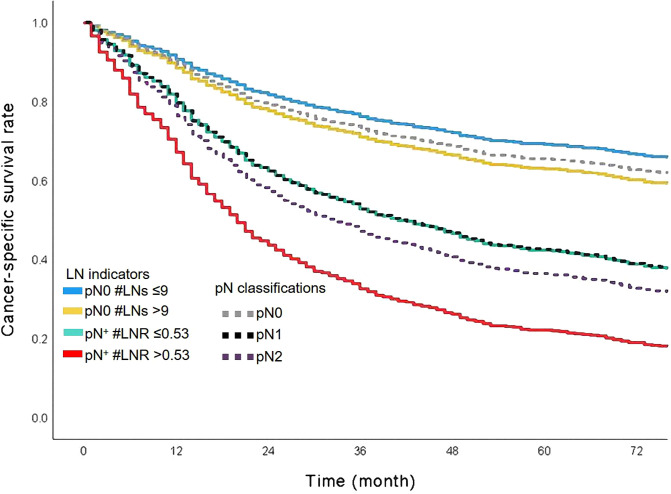
The combined survival curves based on pN classification and LN indicator. LN, lymph node.

N classification was a significant prognostic factor in this cohort (Figure 4). Patients with pN2 classification had worse survival than patients with classification pN0 or pN1. The 5-year CSS rates of classification pN0, pN1, or pN2 were 63.0%, 37.0%, and 33.0%, respectively. We added the survival curves of LN indicators into the same figure to further investigate the stratified difference between N classification and LN indicator. The LN indicator identified a group of patients with the worst survival in this cohort (Figure 4, survival curve in red). The LN indicator had a much better predictive ability over N classification in this cohort (LN indicator: C-index = 0.615, SE  =  0.011; N classification: C-index = 0.602, SE = 0.01, *P* = 0.001).

### Sub-Group Analysis for LN Indicator

**Figure 5:**
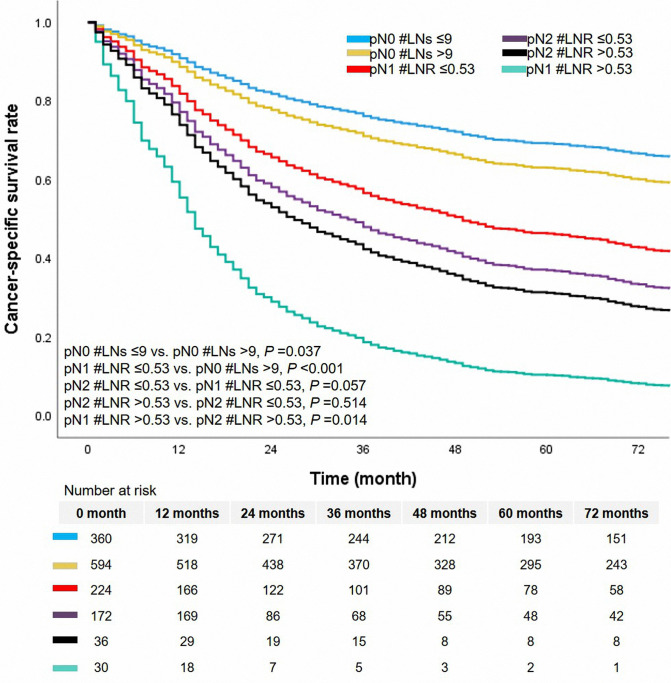
The survival curves of sub-group analysis based on LN indicator. LN, lymph node.

In the cohort of pN1 classification, cases with pN1 #LNR ≤0.53 had a more improved survival than those with pN1 #LNR >0.53 (unadjusted HR = 2.628, 95% CI, 1.695–4.074, *P* < 0.001, Figure 5). The median survival time was 14.0 months (95% CI, 12.1–15.9 months) and 49.0 months (95% CI, 35.4–62.6 months) in the group with pN1 #LNR >0.53 and pN1 #LNR ≤0.53, respectively. However, there was no significant prognostic difference between the cohort with pN2 #LNR >0.53 and pN2 #LNR ≤0.53 (*P* = 0.514). Of note, the cases with pN1 #LNR >0.53 had a decreased prognosis than the entire cohort of pN2 classification (all *P* < 0.05).

## Discussion

In this study, we used a large sample-size cohort to explore the effect of LNs and LNR on pN classification in ASC patients after lobectomy. A composite LN indicator was developed successfully. The results of univariable and multivariable analyses revealed that the LN indicator could be a significant prognostic factor. LN indicators included pN0 #LNs ≤9, pN0 #LNs >9, pN^+^ #LNR ≤0.53 (pN1 #LNR ≤0.53 and pN2 #LNR ≤0.53), and pN^+^ #LNR >0.53 (pN1 #LNR >0.53 and pN2 #LNR >0.53). The level of LNs stratified the patients without metastasis of lymph nodes. Cases with pN^+^ #LNR >0.53 had the worst survival in the entire cohort. We further used the sub-group analysis to uncover that cases with pN1 #LNR ≤0.53 had a more improved survival than those with pN1 #LNR >0.53 in the cohort of pN1 classification. Interestingly, there was no significant prognostic difference between the cohort with pN2 #LNR >0.53 and pN2 #LNR ≤0.53. The reason for this phenomenon might be due to the insufficient sample size of the pN2 cohort. Of note, the cases with pN1 #LNR >0.53 had a decreased prognosis than the entire cohort of pN2 classification. Therefore, we propose that LNs and LNR may be Supplementary information on pN classification and require attention.

Tumor grade was served as a prognostic factor in non-small cell lung cancer patients, according to previous studies (16, 17). However, previous studies were mainly aimed at the squamous cell carcinoma or adenocarcinoma population, and there is a lack of research on the effect of tumor differentiation on the prognosis of ASC patients. The results from a study by Filosso et al. found that the tumor grading had no meaningful impact on the prognosis of ASC patients (9). In the present study, we also found that patients with poor-undifferentiated grading did not have decreased survival outcomes than patients with well-moderate grading after adjusting for other confounders. Regrettably, in some studies involving ASC, information on the degree of tumor differentiation was lacking (18, 19). Therefore, the effect of tumor differentiation degree on the prognosis of ASC patients still needs further study.

The LNs could be a significant indicator to estimate the prognosis of ASC patients. However, the outcomes of a study from Wang et al. found that LNs did not influence the prognosis of ASC patients with stages I-IIIB (18). They analyzed the information about 256 ASC patients after surgery. LNs, as a continuous variable, was selected to enter into the Cox regression in their study. The sample size of their study was not large enough. Another study from Li et al. also treated LNs as a continuous variable; however, in the results of multivariable analysis, LNs was confirmed as an independent prognostic factor (20). The research from Li et al. included 988 patients with stages I-II. The differences between the above two studies were the selection of combined stage and the scale of sample size. The present study revealed that patients with LNs >9 had an improved survival than patients with LNs ≤9. Our findings were similar as the results from the study by Li et al., also uncovered that patients could benefit from a large harvest of lymph node dissection. Therefore, we suggest that surgeons dissect enough lymph nodes during the operation. However, there were some issues with the cutoff point of LNs. For example, different sample sizes and observational cohorts lead to different cutoff values (21, 22). Thus, extensive sample data and defined study populations are still needed to make the cutoff values of LNs more stable.

LNR may reflect the patient’s tumor burden to some extent. Previous reports confirmed that LNR could be a predictive tool to evaluate the prognosis of non-small cell lung cancer patients (11, 13, 14, 23–25). High-level LNR might be associated with the poor survival of those patients. While those previous studies focused on non-small cell lung cancer, our study focused on ASC patients. In the present study, we also obtain similar results to the abovementioned studies. Patients with LNR >0.53 had a much poorer prognosis than patients with LNR ≤0.53. However, the cutoff value of LNR faces similar problems as LNs, and still depends on the sample size and the choice of the study population. Therefore, we suggest that patients’ lymph node metastatic status should be further confirmed, and a larger sample size should be obtained to make the cutoff value more reliable.

The present study has some drawbacks. First, we were also unable to obtain detailed information about the station of the lymph node; therefore, we could not perform the analyses to investigate the effect of lymph-node station dissection on the patients’ prognoses. Second, the treatment sequence was unknown. Thus, the impact of LN indicators on prognoses of patients with neoadjuvant therapy or adjuvant therapy was not analyzed in our research. Third, because the present research belongs to a retrospective study, selection bias is inevitable. In our study, the distribution of N or T classification was not balanced. Finally, the high-level LNs or low-level LNR had survival advantages, but this does not mean they could deny receiving adjuvant treatment. We need more studies to confirm our findings.

## Conclusions

We explored clinicopathological factors affecting prognosis in resected lung ASC patients. Besides, the LN indicator was confirmed to be played an essential role in affecting the survival rate in ASC patients. The high-level LNs or low-level LNR might be corelated to improved survival outcomes.

## Data Availability

The raw data supporting the conclusions of this article will be made available by the authors, without undue reservation.
