# Supplementary material for: The Effect of Examined Lymph Nodes and Lymph Node Ratio on Pathological Nodal Classification in the Lung Adenosquamous Carcinoma After Lobectomy
Source: Front Surg. 2022 Jun 9;9:909810. doi: 10.3389/fsurg.2022.909810 (PMC9218197; doi:10.3389/fsurg.2022.909810)
Supplement: Supplementary file 3 [file Table_3_v1.docx]

| > library(survminer)  > library(survival)  > library(compareC)  > mydata<-read.csv(file.choose())  > mydata<- na.omit(mydata)  > dd <- datadist(mydata)  Error in datadist(mydata) : could not find function "datadist"  > options(datadist="dd")  > cox_model1 <- coxph(Surv(Time, CSS) ~ Nclassification, data=mydata)  > summary(cox_model1)  Call:  coxph(formula = Surv(Time, CSS) ~ Nclassification, data = mydata)  n= 1416, number of events= 672  coef exp(coef) se(coef)  Nclassification 0.4690 1.5985 0.0468  z Pr(>\|z\|)  Nclassification 10.02 <2e-16 ***  ---  Signif. codes:  0 ‘***’ 0.001 ‘**’ 0.01 ‘*’ 0.05 ‘.’ 0.1 ‘ ’ 1  exp(coef) exp(-coef)  Nclassification 1.598 0.6256  lower .95 upper .95  Nclassification 1.458 1.752  Concordance= 0.602 (se = 0.01 )  Likelihood ratio test= 90.01 on 1 df, p=<2e-16  Wald test = 100.4 on 1 df, p=<2e-16  Score (logrank) test = 105.3 on 1 df, p=<2e-16  > cox_model2 <- coxph(Surv(Time, CSS) ~ LNindicator, data=mydata)  > summary(cox_model2)  Call:  coxph(formula = Surv(Time, CSS) ~ LNindicator, data = mydata)  n= 1416, number of events= 672  coef exp(coef) se(coef) z  LNindicator 0.48750 1.62825 0.04706 10.36  Pr(>\|z\|)  LNindicator <2e-16 ***  ---  Signif. codes:  0 ‘***’ 0.001 ‘**’ 0.01 ‘*’ 0.05 ‘.’ 0.1 ‘ ’ 1  exp(coef) exp(-coef) lower .95  LNindicator 1.628 0.6142 1.485  upper .95  LNindicator 1.786  Concordance= 0.615 (se = 0.011 )  Likelihood ratio test= 105.7 on 1 df, p=<2e-16  Wald test = 107.3 on 1 df, p=<2e-16  Score (logrank) test = 109.2 on 1 df, p=<2e-16  > anova(cox_model1,cox_model2)  Analysis of Deviance Table  Cox model: response is Surv(Time, CSS)  Model 1: ~ Nclassification  Model 2: ~ LNindicator  loglik Chisq Df P(>\|Chi\|)  1 -4469.0  2 -4461.1 15.711 0 < 2.2e-16 ***  ---  Signif. codes:  0 ‘***’ 0.001 ‘**’ 0.01 ‘*’ 0.05 ‘.’ 0.1 ‘ ’ 1 |
| --- |
|  |
| \| > \| \| --- \| |
