# Supplementary material for: The Effect of Examined Lymph Nodes and Lymph Node Ratio on Pathological Nodal Classification in the Lung Adenosquamous Carcinoma After Lobectomy
Source: Front Surg. 2022 Jun 9;9:909810. doi: 10.3389/fsurg.2022.909810 (PMC9218197; doi:10.3389/fsurg.2022.909810)
Supplement: Supplementary file 4 [file Table_4_v1.docx]

> library(survival)

> library(survminer)

> rm(list = ls())

> mydata<-read.csv(file.choose())

> str(mydata)

'data.frame': 970 obs. of 47 variables:

$ Sex : int 1 1 0 1 1 0 0 1 0 0 ...

$ Year.of.diagnosis : int 2014 2013 2010 2006 2011 2013 2004 2007 2004 2004 ...

$ Race : int 1 1 1 2 1 1 1 1 1 2 ...

$ Surgery : int 2 2 2 2 2 2 2 2 2 2 ...

$ Radiotherapy : int 0 0 0 0 0 0 0 0 1 0 ...

$ Chemotherapy : int 0 1 0 0 0 0 0 0 1 0 ...

$ CSS : int 1 0 1 0 0 1 0 0 1 1 ...

$ Time : int 14 70 65 135 89 28 169 34 81 24 ...

$ OS : int 1 0 1 1 0 1 1 1 1 1 ...

$ Marital : int 0 1 0 1 1 1 1 0 1 1 ...

$ Grade : int 2 2 3 3 3 3 3 2 5 2 ...

$ Age : int 1 0 1 1 1 1 0 1 1 1 ...

$ LNs : int 7 12 1 6 17 13 3 7 19 7 ...

$ PLN : int 0 0 0 0 0 0 0 0 0 0 ...

$ Nstage : chr "N0" "N0" "N0" "N0" ...

$ T.stage : chr "T2a" "T2b" "T1" "T1" ...

$ Derived.AJCC.M..6th.ed..2004.2015. : chr "M0" "M0" "M0" "M0" ...

$ Patient.ID : int 359120 449445 753562 774188 778774 916035 917271 943653 952512 953217 ...

$ Origin.recode.NHIA..Hispanic..Non.Hisp. : chr "Non-Spanish-Hispanic-Latino" "Non-Spanish-Hispanic-Latino" "Non-Spanish-Hispanic-Latino" "Non-Spanish-Hispanic-Latino" ...

$ Primary.Site...labeled : chr "C34.3-Lower lobe, lung" "C34.1-Upper lobe, lung" "C34.1-Upper lobe, lung" "C34.1-Upper lobe, lung" ...

$ Histologic.Type.ICD.O.3 : int 8560 8560 8560 8560 8560 8560 8560 8560 8560 8560 ...

$ ICD.O.3.Hist.behav..malignant : chr "8560/3: Adenosquamous carcinoma" "8560/3: Adenosquamous carcinoma" "8560/3: Adenosquamous carcinoma" "8560/3: Adenosquamous carcinoma" ...

$ RX.Summ..Surg.Prim.Site..1998.. : int 33 30 30 33 33 33 33 33 33 33 ...

$ CS.tumor.size..2004.2015. : int 33 43 10 24 31 10 20 15 35 20 ...

$ CS.extension..2004.2015. : int 430 100 100 100 100 420 100 100 650 100 ...

$ CS.lymph.nodes..2004.2015. : int 0 0 0 0 0 0 0 0 0 0 ...

$ CS.mets.at.dx..2004.2015. : int 0 0 0 0 0 0 0 0 0 0 ...

$ Derived.AJCC.Stage.Group..6th.ed..2004.2015. : chr "IB" "IB" "IA" "IA" ...

$ Derived.AJCC.T..6th.ed..2004.2015. : chr "T2" "T2" "T1" "T1" ...

$ Age.recode.with..1.year.olds : chr "65-69 years" "60-64 years" "70-74 years" "75-79 years" ...

$ Sequence.number : chr "2nd of 2 or more primaries" "2nd of 2 or more primaries" "2nd of 2 or more primaries" "2nd of 2 or more primaries" ...

$ First.malignant.primary.indicator : chr "No" "No" "No" "No" ...

$ Primary.by.international.rules : chr "Yes" "Yes" "Yes" "Yes" ...

$ Record.number.recode : int 2 1 2 2 2 2 1 2 1 1 ...

$ Total.number.of.in.situ.malignant.tumors.for.patient : int 2 2 5 2 2 3 1 2 1 1 ...

$ Total.number.of.benign.borderline.tumors.for.patient : int 0 0 0 0 0 0 0 0 0 0 ...

$ Type.of.Reporting.Source : chr "Hospital inpatient/outpatient or clinic" "Hospital inpatient/outpatient or clinic" "Hospital inpatient/outpatient or clinic" "Hospital inpatient/outpatient or clinic" ...

$ CS.site.specific.factor.1..2004.2017.varying.by.schema.: int 0 0 0 988 0 0 988 988 988 988 ...

$ CS.site.specific.factor.2..2004.2017.varying.by.schema.: chr "20" "999" "0" "Blank(s)" ...

$ CS.site.specific.factor.3..2004.2017.varying.by.schema.: chr "Blank(s)" "Blank(s)" "Blank(s)" "Blank(s)" ...

$ CS.site.specific.factor.4..2004.2017.varying.by.schema.: chr "Blank(s)" "Blank(s)" "Blank(s)" "Blank(s)" ...

$ COD.to.site.recode : chr "Lung and Bronchus" "Alive" "Lung and Bronchus" "Colon excluding Rectum" ...

$ RX.Summ..Scope.Reg.LN.Sur..2003.. : chr "4 or more regional lymph nodes removed" "4 or more regional lymph nodes removed" "1 to 3 regional lymph nodes removed" "1 to 3 regional lymph nodes removed" ...

$ RX.Summ..Surg.Oth.Reg.Dis..2003.. : chr "None; diagnosed at autopsy" "None; diagnosed at autopsy" "None; diagnosed at autopsy" "None; diagnosed at autopsy" ...

$ X : logi NA NA NA NA NA NA ...

$ X.1 : logi NA NA NA NA NA NA ...

$ Reason.no.cancer.directed.surgery : chr "Surgery performed" "Surgery performed" "Surgery performed" "Surgery performed" ...

> cutoff<-surv_cutpoint(mydata,

+ time="Time",

+ event="CSS",

+ variables=c("LNs"))

> summary(cutoff)

cutpoint statistic

LNs 9 2.083362

> plot(cutoff,

+ "LNR",

+ palette = "lancet")

Error in `[.data.frame`(data, , variables, drop = FALSE) :

选择了未定义的列

> plot(cutoff,

+ "LNs",

+ palette = "lancet")

$LNs

> mydata<-read.csv(file.choose())

> str(mydata)

'data.frame': 462 obs. of 48 variables:

$ Sex : int 1 1 0 1 1 0 0 0 1 0 ...

$ Year.of.diagnosis : int 2014 2004 2013 2012 2008 2010 2004 2005 2005 2009 ...

$ Race : int 1 1 1 1 2 1 2 1 2 1 ...

$ Surgery : int 2 2 2 2 2 2 2 2 2 2 ...

$ Radiotherapy : int 1 1 1 0 0 0 1 1 0 0 ...

$ Chemotherapy : int 1 1 1 0 0 0 1 0 1 1 ...

$ CSS : int 0 1 1 1 0 1 1 1 1 0 ...

$ Time : int 40 20 41 38 2 8 98 21 12 77 ...

$ OS : int 1 1 1 1 1 1 1 1 1 1 ...

$ Marital : int 1 0 0 0 0 1 0 1 2 0 ...

$ Grade : int 5 3 3 3 3 2 3 2 2 3 ...

$ Age : int 1 0 1 1 1 1 0 1 0 1 ...

$ LNs : int 35 14 13 14 24 8 3 23 5 22 ...

$ PLN : int 3 6 6 1 6 3 1 7 1 1 ...

$ LNR : num 0.0857 0.4286 0.4615 0.0714 0.25 ...

$ Nstage : chr "N2" "N2" "N2" "N1" ...

$ T.stage : chr "T1" "T3" "T2a" "T1" ...

$ Derived.AJCC.M..6th.ed..2004.2015. : chr "M0" "M0" "M0" "M0" ...

$ Patient.ID : int 799771 800371 859414 883685 888077 929199 948158 973501 975353 1007279 ...

$ Origin.recode.NHIA..Hispanic..Non.Hisp. : chr "Non-Spanish-Hispanic-Latino" "Non-Spanish-Hispanic-Latino" "Spanish-Hispanic-Latino" "Non-Spanish-Hispanic-Latino" ...

$ Primary.Site...labeled : chr "C34.3-Lower lobe, lung" "C34.3-Lower lobe, lung" "C34.1-Upper lobe, lung" "C34.3-Lower lobe, lung" ...

$ Histologic.Type.ICD.O.3 : int 8560 8560 8560 8560 8560 8560 8560 8560 8560 8560 ...

$ ICD.O.3.Hist.behav..malignant : chr "8560/3: Adenosquamous carcinoma" "8560/3: Adenosquamous carcinoma" "8560/3: Adenosquamous carcinoma" "8560/3: Adenosquamous carcinoma" ...

$ RX.Summ..Surg.Prim.Site..1998.. : int 33 33 33 30 33 33 33 33 33 30 ...

$ CS.tumor.size..2004.2015. : int 16 51 24 20 50 38 12 30 50 25 ...

$ CS.extension..2004.2015. : int 115 100 430 100 300 100 100 450 100 100 ...

$ CS.lymph.nodes..2004.2015. : int 200 200 200 100 200 200 200 200 200 100 ...

$ CS.mets.at.dx..2004.2015. : int 0 0 0 0 0 0 0 0 0 0 ...

$ Derived.AJCC.Stage.Group..6th.ed..2004.2015. : chr "IIIA" "IIIA" "IIIA" "IIA" ...

$ Derived.AJCC.T..6th.ed..2004.2015. : chr "T1" "T2" "T2" "T1" ...

$ Age.recode.with..1.year.olds : chr "70-74 years" "60-64 years" "70-74 years" "80-84 years" ...

$ Sequence.number : chr "2nd of 2 or more primaries" "2nd of 2 or more primaries" "2nd of 2 or more primaries" "2nd of 2 or more primaries" ...

$ First.malignant.primary.indicator : chr "Yes" "No" "No" "No" ...

$ Primary.by.international.rules : chr "Yes" "Yes" "Yes" "Yes" ...

$ Record.number.recode : int 2 2 2 2 1 2 1 1 1 2 ...

$ Total.number.of.in.situ.malignant.tumors.for.patient : int 3 2 2 2 2 2 1 1 1 3 ...

$ Total.number.of.benign.borderline.tumors.for.patient : int 0 0 0 0 0 0 0 0 0 0 ...

$ Type.of.Reporting.Source : chr "Hospital inpatient/outpatient or clinic" "Hospital inpatient/outpatient or clinic" "Hospital inpatient/outpatient or clinic" "Hospital inpatient/outpatient or clinic" ...

$ CS.site.specific.factor.1..2004.2017.varying.by.schema.: int 0 988 0 0 988 0 988 988 988 988 ...

$ CS.site.specific.factor.2..2004.2017.varying.by.schema.: chr "0" "Blank(s)" "20" "0" ...

$ CS.site.specific.factor.3..2004.2017.varying.by.schema.: chr "Blank(s)" "Blank(s)" "Blank(s)" "Blank(s)" ...

$ CS.site.specific.factor.4..2004.2017.varying.by.schema.: chr "Blank(s)" "Blank(s)" "Blank(s)" "Blank(s)" ...

$ COD.to.site.recode : chr "Stomach" "Lung and Bronchus" "Lung and Bronchus" "Lung and Bronchus" ...

$ RX.Summ..Scope.Reg.LN.Sur..2003.. : chr "4 or more regional lymph nodes removed" "Sentinel node biopsy and lym nd removed same/unstated time" "4 or more regional lymph nodes removed" "4 or more regional lymph nodes removed" ...

$ RX.Summ..Surg.Oth.Reg.Dis..2003.. : chr "None; diagnosed at autopsy" "None; diagnosed at autopsy" "None; diagnosed at autopsy" "None; diagnosed at autopsy" ...

$ X : logi NA NA NA NA NA NA ...

$ X.1 : logi NA NA NA NA NA NA ...

$ Reason.no.cancer.directed.surgery : chr "Surgery performed" "Surgery performed" "Surgery performed" "Surgery performed" ...

> cutoff<-surv_cutpoint(mydata,

+ time="Time",

+ event="CSS",

+ variables=c("LNR"))

> summary(cutoff)

cutpoint statistic

LNR 0.5263158 3.200659

> plot(cutoff,

+ "LNs",

+ palette = "lancet")

Error in `[.data.frame`(data, , variables, drop = FALSE) :

选择了未定义的列

> plot(cutoff,

+ "LNR",

+ palette = "lancet")

$LNR
